# Production and validation of an educational video on the use of the Z-Track Technique

**DOI:** 10.1590/0034-7167-2022-0439

**Published:** 2023-03-17

**Authors:** Patricia Magnabosco, Simone de Godoy, Isabel Amélia Costa Mendes, Maria Beatriz Guimarães Raponi, Bruna Francielle Toneti, Leila Maria Marchi-Alves

**Affiliations:** I Universidade de São Paulo. Ribeirão Preto, São Paulo, Brazil; II Universidade Federal de Uberlândia. Uberlândia, Minas Gerais, Brazil

**Keywords:** Injections, Intramuscular, Validation Study, Instructional Film and Video, Video-Audio Media, Education, Nursing., Inyecciones Intramusculares, Estudio de Validación, Película y Video Educativos, Medios Audiovisuales, Educación en Enfermería., Injeções Intramusculares, Estudos de Validação, Filmes e Vídeos Educativos, Mídia Audiovisual, Educação em Enfermagem.

## Abstract

**Objective:**

to create and validate an educational video on intramuscular drug administration using the Z-track technique.

**Methods:**

the Delphi Technique was used to validate the script. PhDs in Nursing and PhDs in Social Communication with experience in the production of educational videos participated in the process. After editing, the video was validated by three nursing professors and assessed by students of the undergraduate nursing program at a public university.

**Results:**

the video was validated by the examiners with 100% agreement in three rounds for script validation and in two for video validation after editing. The duration of the video was 9 minutes.

**Conclusion:**

after validation by the examiners, students assessed the video and considered it suitable for learning. We This video is expected to aid in the training of nursing professionals and the enhancement of patient care.

## RESUMO

**Objetivo:**

construir e validar um vídeo educativo sobre a administração de medicamentos por via intramuscular, com utilização da Técnica em Z.

**Métodos:**

utilizou-se a Técnica Delphi para a validação do roteiro. Participaram do processo doutores em enfermagem e doutores em comunicação social com experiência na produção de vídeos educativos. Após a edição, o vídeo foi validado por três docentes de enfermagem e avaliado por estudantes do Curso de Graduação em Enfermagem de uma universidade pública.

**Resultados:**

o vídeo foi validado pelos juízes com 100% de concordância ocorrida em três rodadas para validação do roteiro e duas para validação do vídeo após a edição. A duração do vídeo foi de 9 minutos.

**Conclusão:**

Após a validação pelos juízes, estudantes avaliaram o vídeo e o consideraram adequado para o aprendizado. Espera-se que esse vídeo possa contribuir para a formação do profissional de enfermagem e melhoria da assistência ao paciente.

## RESUMEN

**Objetivo:**

construir y validar un vídeo educativo sobre la administración intramuscular de fármacos, utilizando la Técnica Z.

**Métodos:**

se utilizó la técnica Delphi para validar el guion. Participaron en el proceso dos doctores en enfermería y dos doctores en comunicación social con experiencia en la producción de vídeos educativos. Tras su edición, el vídeo fue validado por tres docentes de enfermería y evaluado por estudiantes del curso de licenciatura en enfermería de una universidad pública.

**Resultados:**

el vídeo fue validado por los expertos con un 100% de acuerdo en tres rondas para la validación del guion y dos para la validación del vídeo después de la edición. La duración del vídeo fue de 9 minutos.

**Conclusión:**

tras la validación por parte de los expertos, los estudiantes evaluaron el vídeo y lo consideraron adecuado para el aprendizaje. Se espera que este vídeo pueda contribuir a la formación de los profesionales de enfermería y mejorar la asistencia a los pacientes.

## INTRODUCTION

The nursing team is commonly responsible for intramuscular (IM) drug administration, which requires a broad knowledge base with solid technical-scientific foundations^(1)^.

Although the IM route has advantages over the oral and other parenteral routes, such as the potential to increase drug absorption and bioavailability^(2)^, there are significant risks associated with drug administration via this route, particularly when the procedure is performed incorrectly or negligently. In addition to pain and anxiety, possible harms include the occurrence of sterile abscess, infection, tissue irritation, periostitis, fibrosis, muscle contracture, necrosis, gangrene, and hemorrhage^(3-4)^. Other adverse events associated with the procedure include decreased drug efficacy, lower drug absorption rate, and delayed drug action, particularly when the injection site is incorrectly delimited^(5-6)^.

Some authors emphasize that IM injection injuries can be prevented with the proper techniques^(7)^. The Z technique or Z-track technique^(8-9)^, which reduces the risk of administering the drug to a tissue other than the muscle, thus preventing loss or extravasation of solution after its introduction, is one of the techniques capable of minimizing the risks and injuries associated with the procedure^(10-11)^.

Despite the literature demonstrating that the Z-track technique is the most appropriate for drug administration via the IM route, it is rarely used in practice. According to studies conducted with nursing professionals, more than half of the team members had never used the Z-track technique^(12-13)^; according to other authors, a significant number of nursing professionals reported not knowing the procedure and only a few performed it routinely^(13-14)^.

This impediment to the use of the Z-track technique in the daily routine of health services, particularly by nursing professionals, may be related to deficiencies in the professional training process. It is advised that the teaching of skills-based techniques be coordinated with evidence-based applications and enhanced learning technologies^(15)^.

In this context, educational resources should be developed to improve the safety of drug administration via the IM route, as the occurrence of harm can be reduced by acquisition of scientific knowledge and technical skills. From this perspective, traditional learning methods and skill laboratories should be enhanced with the use of information and communication technologies^(1)^.

Digital information and communication technologies (DICT) integrate pedagogical practices through digital resources, resulting in the creation of new, innovative, and collaborative learning spaces with the goal of promoting autonomy and interactivity in the teaching-learning process^(16-17)^. Educational videos stand out among the various teaching-learning tools that have been used for this purpose.

Educational videos can represent a level of sophistication in the teaching-learning relationship because it can capture the public’s attention while also arousing their curiosity about the topics addressed, stimulate creativity, and develop educational practice in a straightforward and objective manner^(18)^. Furthermore, it has several advantages, such as being a practical method, having a good cost benefit, allowing information to be easily understood, and allowing scenes to be watched multiple times^(19-20)^. It can be used in a variety of teaching settings, including classrooms, simulation laboratories, and distance education, to promote the acquisition of new skills and improve the learning process of students^(21)^.

## OBJECTIVE

To produce and validate an educational video on IM drug administration using the Z-track technique.

## METHODS

### Ethical aspects

The research project was submitted to a human research ethics committee and data collection began after the project was approved.

### Study type, period, and location

This is a methodological study that was conducted from September 2021 to July 2022. The steps for producing the video were as follows: pre-production (script development), production (video recording), and post-production (video editing)^(22)^. Before the production stage, the script was validated by specialists, and after the post-production stage, the video was validated by specialists and evaluated by the target population^(23-24)^. The video was produced in the practice simulation center of a public teaching institution in the state of São Paulo, and it was assessed by the target population at a public university in the state of Minas Gerais.

### Population; inclusion and exclusion criteria

The inclusion criteria for content validation were being a professor who taught the Fundamentals of Nursing course discipline or an assistant nurse with experience in IM drug administration for content validation, and the inclusion criterion for technical validation was being a social communication professor with experience in producing educational videos. Experts who did not hold at least a doctorate were excluded.

Three professors and two nurses participated in the content validation, and two professors participated in the technical validation.

An email invitation was sent to the experts outlining the objectives of the study and providing information on the material to be evaluated. The email included an informed consent form. After the examiners agreed to participate in the study and signed the informed consent form, the initial version of the video script and the instruments for content^(25)^ and technical^(26)^ validation were sent, along with a characterization questionnaire on the participants’ qualification and professional background^(25,27)^. All instruments provided space for the examiners to record their thoughts and/or suggestions about each item evaluated.

The content validation instrument examined six categories: objectives, content, relevance, environment, verbal language, and topic inclusion. Each item in the six categories was assessed by a four-point Likert scale. (4, strongly agree; 3, agree; 2, disagree; 1, strongly disagree)^(25)^.

An instrument containing the following categories was used for audiovisual technical validation: concept of the idea; dramatic construction (opening, conflict, development, climax, conclusion); rhythm (evolution of dramatic moments, types of scenes); characters (motivation, credibility, interaction); dramatic potential, dialogues (dramatic time), visual style (aesthetics), referring audience, production estimate, functionality, usability, efficiency, and final analysis results. Each item was rated as: 4. Excellent; 3. Very good; 2. Good; 1. Regular; 0. Less regular. The examiner chose one of the four options as the final result: 4. Approved, 3. Approved with modifications, 2. Rejected with positive aspects, or 1. Rejected^(26)^.

The Delphi technique, which has been used in other studies, was used to validate the video script^(19,21,24)^. The Delphi technique is an expert evaluation method aimed at obtaining successive suggestions, criticisms, and opinions on items in a questionnaire. Each round draws on information and comments from previous rounds, which are analyzed and integrated into the next round to generate new suggestions, with the goal of reaching a consensus among the examiners^(28)^.

The Delphi technique has several advantages: it can be conducted via e-mail and does not require specialists to meet in person; the anonymity of the process prevents an opinionated examiner from dominating the process and influencing the opinion of the group; it allows the accumulation of different opinions reaching consensus among experts^(28)^.

For this validation stage, the percentage of expert agreement was set at 100%^(21)^.

The results were analyzed descriptively using average scores and presented in tables.

The final version of the script was created after analyzing the data and reaching a consensus among the experts, both in terms of content validity and audiovisual communication.

The educational video was recorded in the practice simulation center of a public educational institution in the state of São Paulo. Following the validated script, the scenes were filmed repeatedly over a four-hour period. Two participants took part, one acting as a nurse and the other as a patient.

Following the completion of the filming, text, image, and sound graphic animation resources were included. The video was narrated by a professional with voiceover experience and was recorded using the “recorder” application available on the iPhone 7® with iOS 15.5 operating system, and images were edited using Adobe Premiere and Adobe Photoshop by communication technicians who were experienced in video development. It took 25 days of editing to cover the content of the validated script, adjust the image, audio, and soundtrack sequences, and make the necessary adaptations for the target population. The video was sent to three Nursing Fundamentals professors for face and content validation after the sixth version.

The instrument with six categories, related to functionality, usability, efficiency, audiovisual technique, environment, and procedure, was used to validate the educational video. Each item of the categories was rated as “4. strongly agree,” “3. agree,” “2. disagree,” and “1. strongly disagree”^(25)^.

The data analysis technique and the criteria for agreement values among the experts were the same as in the script validation stage.

### Video assessment by the target population

The video was assessed by fifteen students from a non-probabilistic sample of the undergraduate nursing program at a public university in the state of Minas Gerais.

Some authors recommend six to twenty subjects for technology/instrument validation^(29)^. Choosing an odd number of participants avoids a tie in dubious answers and questions^(30)^.

The inclusion criteria were as follows: a) being regularly enrolled in an undergraduate nursing program; b) having completed the theoretical and practical modules of the Fundamentals of Nursing discipline; and c) being 18 years old or older. Students enrolled in a special learning regime and those who had not been approved for the Fundamentals of Nursing discipline were excluded.

The students were invited at random via an electronic address and in descending order from the most advanced periods of the program. They were taken to an appropriate and silent environment to watch the educational video individually through a laptop after consenting to participate in the study and signing the informed consent form. The date and time were chosen based on the students’ availability and that of the room.

After watching the video, the students were given the educational video assessment tool by the target population called “Assistive Technology Assessment Questionnaire” for their assessment. This validated tool consists of four items to verify the understanding of educational assistive technologies: objectives, clarity, relevance, and interactivity. Students assigned a score from 0 to 2 for each attribute, with (0) Inadequate, (1) Partially adequate, (2) Adequate, and a space was provided for the students to comment, criticize, or suggest the aspects they considered positive or negative, if applicable^(31)^.

### Statistical analysis

The results of the assistive technology assessment questionnaire were organized based on the synthesis of the responses, and they were analyzed descriptively using mean, median, standard deviation, maximum and minimum values, and the Statistical Package for the Social Sciences software - SPSS for Windows, version 25.0.

## RESULTS

The video script was validated by seven experts, five from the nursing field and two from social communication. The experts were professors of the Fundamentals of Nursing discipline, nurses, and professors of social communication with experience in the development of educational videos, aged 31 to 65 years old (mean of 41.3 years), all with a PhD. Professional experience in the area ranged from 8 to 20 years, with a mean of 13.3 years.

Items that did not achieve the maximum value (score 4 or “strongly agree”) were reformulated based on the suggestions of the experts, and a new script was submitted for reevaluation in subsequent rounds.

The video script was validated after three rounds of content validation and two rounds of technical validation over a four-month period.

Table 1 shows the mean scores obtained in each item of the content validation tools.

**Table 1:** Mean scores of the items in each category of the adapted tool^*^ used to validate the script’s content in the three rounds, Ribeirão Preto, São Paulo, Brazil, 2021-2022

Items by category	Mean scores		
	Round 1	Round 2	Round 3
Objectives			
1. The objectives are consistent with nursing practice.	4	4	4
2. The objectives are consistent with the objectives proposed in the study.	4	4	4
3. The objectives are achievable.	4	4	4
Contents			
1. The content presented in the script corresponds to the objectives proposed in the study.	3.8	4	4
2. The content facilitates the teaching-learning process on the subject.	4	4	4
3.The content allows understanding of the topic.	4	4	4
4. The content follows a logical sequence.	4	4	4
5. The content addresses all the steps necessary to administer drugs via the intramuscular route using the Z technique in an orderly manner.	4	4	4
6. The content includes all the materials necessary to administer drugs via the intramuscular route using the Z-track technique.	3.8	4	4
7. The information in the script is correct.	3.4	3,8	4
Relevance			
1. The images and scenes illustrate important aspects for drug administration via the intramuscular route using the Z-track technique.	3.8	4	4
2. The images and scenes are relevant to aid the user to administer drugs with better performance.	3.8	4	4
3. The images and scenes allow the transfer and generalization of learned content to different contexts.	3.6	4	4
Environment			
1.The scenario is suitable for video broadcast.	4	4	4
2. The scenario is suitable for learning the subject.	4	4	4
Verbal language			
1. The verbal language used in the script is accessible to the target audience.	4	4	4
2. Verbal language is easy to understand.	4	4	4
Topic inclusion			
1. Objectives of the educational video.	4	4	4
2. Brief history of intramuscular drug administration using the Z-track technique.	4	4	4
3. Purpose of intramuscular drug administration using the Z-track technique.	4	4	4
4. Objectives of intramuscular drug administration using the Z-track technique.	4	4	4
5. Description of the steps of the procedure for intramuscular drug administration using the Z-track technique.	4	4	4

* *Ferreira MVF. Dressing of central venous catheters: supports for nursing teaching and care. Dissertation (Doctoral) - University of São Paulo at Ribeirão Preto College of Nursing, 2013^(25)^*

The experts in the nursing field made the following suggestions in the first round: adding information on the need for traction on the plunger to verify venous return; replacing the seven “rights” framework in drug administration with the nine “rights” framework; changing the terms “prescription” to “medical prescription” and “medication” to “drug”; adding information on packaging disposal in recyclable waste; signaling the anatomical structures in the actor’s (patient) body in order to guide the injection site; correcting the positioning of the non-dominant hand in the figure showing skin traction.

Only the addition of the word “ventrogluteal” to the video title was suggested in the second round.

Only in the first round did the experts make changes to the video script’s technical (audiovisual) validation, namely: adding more images, vignettes, and videographics; revamping the soundtrack to something more rhythmic; including duration time of each scene; dynamizing the action times of each scene; modifying the excessive freezing of scenes; not repeating many types of shooting plans; describing the type of light used in the compositions of the plans; estimating the time of the final product; using an average time of 10 seconds for each scene; using other transition effects (scene freeze) such as fade in, fade out, dry cut, and overlay.

Following script validation and video production, the video validation stage was carried out by three professors of the discipline of Fundamentals of Nursing aged 31 to 42 years, with an average of 35 years and professional experience in the area of 12 years.

Video validation took two rounds. The following suggestions were made in the first round: adjusting the speech/narration with the action in one of the scenes, changing the moment of presentation of the check of two “rights”, form and action of the drug, inserting the information of disinfection with alcohol 70% of the bottleneck of the ampoule at the time of preparation of the drug, and adding speech/narration for better understanding of some steps presented in text form.

The examiners strongly agreed (score equal to 4) with all items of the instrument in the second round, with no new recommendation/suggestion.

Table 2 shows the mean scores for each round.

**Table 2:** Mean scores of the categories of the tool^*^ used for video validation in the two rounds, Ribeirão Preto, São Paulo, Brazil, 2021-2022

Items by category	Mean scores	
	Round 1	Round 2
Functionality		
The video is as an adequate tool for the purpose for which it is intended.	4	4
The video generates positive results in the teaching-learning process on the subject.	4	4
Usability		
The video is easy to use.	4	4
It is easy to learn the theoretical concepts used and their applications.	4	4
It allows the user to easily apply the concepts addressed in nursing practice.	4	4
Efficiency		
The duration of the video (time used) is adequate for the user to learn the content.	4	4
The number of scenes is consistent with the time proposed for the video.	4	4
Audiovisual technique		
The lighting is adequate for watching the scenes.	4	4
The narrator's tone and voice are clear and appropriate.	4	4
The video narration is used efficiently and understandably to the target audience.	4	4
It is possible to return to any part of the scenes when desired.	4	4
Environment		
The video reflects the daily practice of nursing.	4	4
The laboratory environment did not interfere with the fidelity of the IM drug administration procedure using the Z-track technique.	4	4
Procedure		
Objectives of the educational video.	4	4
Brief history of IM drug administration using the Z-track technique.	4	4
Purpose of IM drug administration using the Z-track technique.	4	4
Objectives of IM drug administration using the Z-track technique.	4	4
All the materials used in the procedure were presented.	4	4
The steps of the IM drug administration procedure using the Z-track technique are adequate and could be identified.	3.3	4

* *Ferreira MVF. Dressing of central venous catheters: supports for nursing teaching and care. Dissertation (Doctoral) - University of São Paulo at Ribeirão Preto College of Nursing, 2013^(25)^*

**Table 3:** Measures of central tendency and variability for the items of the tool^*^ used for video assessment by the target audience (n=15), Ribeirão Preto, São Paulo, Brazil, 2021-2022

Attributes	Item		Mean	Standard deviation	Median	Min	Max
1 Interactivity	1	The information content is tailored to your needs.	2.00	0.00	2	2	2
	2	Offers interaction, active involvement in the educational process.	1.73	0.45	2	1	2
	3	Promotes easy access to the topics presented.	2.00	0.00	2	2	2
	4	Provides autonomy to the student in relation to its completion.	1.87	0.35	2	1	2
2 Objectives	5	Stimulates learning about the content addressed.	2.00	0.00	2	2	2
	6	Stimulates learning new concepts.	2.00	0.00	2	2	2
	7	Allows you to search for information without difficulties.	2.00	0.00	2	2	2
	8	Has an attractive presentation strategy.	1.87	0.35	2	1	2
3 Relevance and effectiveness	9	Provides the appropriate and necessary resources for its use.	2.00	0.00	2	2	2
	10	Arouses your interest to use it.	2.00	0.00	2	2	2
	11	Stimulates you to change behaviors.	1.93	0.25	2	1	2
	12	Reproduces the content addressed in different contexts.	1.80	0.41	2	1	2
4 Clarity	13	Presents information in a simple way.	2.00	0.00	2	2	2
	14	Allows you to reflect on the content presented.	2.00	0.00	2	2	2
5 Video as a whole	15	In general, how would you rate the video for learning how to administer drugs via IM using the Z-track technique.	2.00	0.00	2	2	2

* *Guimarães FJ, Carvalho ALRF, Pagliuca LMF. Elaboration and validation of an assistive technology assessment questionnaire. Rev. Eletr. Enf. 2015; 17(2): 302-11^(31)^*

Following the completion of the validation and reaching 100% agreement among the experts, the video was completed with a duration of nine minutes.

Fifteen students from the fourth to tenth periods of the undergraduate nursing program participated in the video evaluation stage, ranging in age from 22 to 46 years, with an average of 25.2 years.

## DISCUSSION

Videos and films have been used extensively as a didactic resource in education over the last few decades. This has been observed in health education, particularly in nursing education, because it brings students closer to real-world service settings through procedure illustrations, promoting the understanding of clinical competences^(32)^.

The video created and validated in this study depicted all steps of a nursing professional in a hospital unit administering drugs via IM in the ventrogluteal region using the Z-track Technique.

Although existing studies consider a proportion of agreement between experts equal to or greater than 80% for video validation^(33)^, we decided to use a proportion of 100% of consensus between experts due to the rigor in the pre-production phase, a criterion also adopted by other studies^(21,24)^.

According to some authors, the duration of educational videos should not exceed 10 to 15 minutes^(19,22)^, because the duration interferes with the students’ level of attention^(34)^. However, there have been reports of videos lasting between 16 and 37 minutes in the literature^(21,24,35)^. The duration of the video in this study was within the limits recommended by the literature.

Three rounds of assessment were required for script validation by experts in the nursing field, two rounds for the examiners in the technical field, and two rounds for the final validation of the video in the post-production stage. One of the precautions to be taken before using the Delphi technique is to avoid excessive rounds, limiting them to a maximum of four^(36)^.

Only suggestions in terms of content and relevance were made of the seven categories addressed in the script validation instrument assessed by nursing experts. The main recommendations focused on the need to better demonstrate the anatomical boundaries of the injection site in the ventrogluteal region.

Using a realistic simulation scenario in health provides a favorable learning environment, assisting in the development of essential competences and skills for professional training^(37)^. Thus, in order to best simulate reality and comply with the experts’ recommendations, the procedure of IM injection using the Z-track technique was performed using the actor’s own body (patient), except at the time of needle insertion, when an anatomical piece created by the researchers was fixed on the delimited area to simulate the injection site.

The main categories addressed by the experts in the field of social communication for the validation of the video script were visual style (aesthetics) and production estimate.

Following the suggestion of the examiners in the first round of video validation, the narration of all steps of the Z-track technique procedure was included to the presentation, which was previously only in text format. Language adequacy in instructional material production is required to improve understanding of the topic and is widely recommended in the production of materials for health education^(38-39)^.

In addition to boring viewers and causing abstraction, the use of confusing language with difficult-to-understand terms and incorrect concepts can impair the performance of the procedure and, as a result, compromise patient safety. Therefore, it is critical to obtain agreement between the examiners and the target population on the concept, clarity, and language used in the video to ensure understanding of its content while making it more appealing^(19)^.

Although all students thought the video was adequate for learning how to administer drugs via IM using the Z-track Technique, the items “offers interaction, active participation in the educational process” and “reproduces the content addressed in different contexts” received the lowest scores from the participants.

The fact that some students considered the video as unfavorable to active participation in the educational process, despite being dynamic, interesting, stimulating, and reflective for learning the content addressed, may be due to the characteristics of the videos, which are aimed at viewers receptive to information in order to demonstrate procedures, as opposed to educational games, which have the potential to provide students with opportunities to participate more actively^(40)^.

The evaluation of the reproduction of the content addressed in different contexts confirmed that the scenario depicted in the video depicts the hospital environment. However, it does not mean that the procedure cannot be replicated in other healthcare settings. All IM injections should be administered using the Z-track technique^(41-43)^.

### Study limitations

The validation process represented a limitation in the development of the study as the content validity was only completed due to the time established to fulfill the research schedule. Despite the study’s limitations, the video was positively assessed by the examiners and the target audience during the validation process, achieving the goal for which it was proposed.

### Contributions to the area

The educational video created in this study potentially collaborates to the training of scholars and health professionals, with the benefits of strengthening the theoretical-practical foundation in the critical educational process aimed at improving the quality of care through educational strategies capable of guiding the achievement of safety goals and promotion of safe care.

This study is expected to serve as a catalyst for further research on the subject, promoting scientific research in the field.

## CONCLUSIONS

Both the examiners and the target audience found the educational video to be valid in terms of content.
